# Cortical Thickness of Brain Areas Beyond Stroke Lesions and Sensory-Motor Recovery: A Systematic Review

**DOI:** 10.3389/fnins.2021.764671

**Published:** 2021-11-03

**Authors:** Anna Maria Cortese, Luisa Cacciante, Anna-Lisa Schuler, Andrea Turolla, Giovanni Pellegrino

**Affiliations:** ^1^Laboratory of Rehabilitation Technologies, San Camillo Istituto di Ricovero e Cura a Carattere Scientifico, Venice, Italy; ^2^Laboratory of Clinical Imaging and Stimulation, San Camillo Istituto di Ricovero e Cura a Carattere Scientifico, Venice, Italy

**Keywords:** stroke, cortical thickness, cortical atrophy, recovery, brain lesion, diaschisis, plasticity, rehabilitation

## Abstract

**Background:** The clinical outcome of patients suffering from stroke is dependent on multiple factors. The features of the lesion itself play an important role but clinical recovery is remarkably influenced by the plasticity mechanisms triggered by the stroke and occurring at a distance from the lesion. The latter translate into functional and structural changes of which cortical thickness might be easy to quantify one of the main players. However, studies on the changes of cortical thickness in brain areas beyond stroke lesion and their relationship to sensory-motor recovery are sparse.

**Objectives:** To evaluate the effects of cerebral stroke on cortical thickness (CT) beyond the stroke lesion and its association with sensory-motor recovery.

**Materials and Methods:** Five electronic databases (PubMed, Embase, Web of Science, Scopus and the Cochrane Library) were searched. Methodological quality of the included studies was assessed with the Newcastle-Ottawa Scale for non-randomized controlled trials and the Risk of Bias Cochrane tool for randomized controlled trials.

**Results:** The search strategy retrieved 821 records, 12 studies were included and risk of bias assessed. In most of the included studies, cortical thinning was seen at the ipsilesional motor area (M1). Cortical thinning can occur beyond the stroke lesion, typically in regions anatomically connected because of anterograde degeneration. Nonetheless, studies also reported cortical thickening of regions of the unaffected hemisphere, likely related to compensatory plasticity. Some studies revealed a significant correlation between changes in cortical thickness of M1 or somatosensory (S1) cortical areas and motor function recovery.

**Discussion and Conclusions:** Following a stroke, changes in cortical thickness occur both in regions directly connected to the stroke lesion and in contralateral hemisphere areas as well as in the cerebellum. The underlying mechanisms leading to these changes in cortical thickness are still to be fully understood and further research in the field is needed.

**Systematic Review Registration:** https://www.crd.york.ac.uk/prospero/display_record.php?ID=CRD42020200539; PROSPERO 2020, identifier: CRD42020200539.

## Introduction

Stroke is the leading cause of disability in western countries, with more than 3 million people left with a disability every year (Dobkin, 2005; Vos et al., 2016).

Stroke lesion triggers a multitude of systemic and cerebral effects, such as neurogenesis, gliogenesis and axonal sprouting, which, together with genetic (e.g., polymorphisms, transcriptome) and environmental factors (e.g., time point and intensity of rehabilitation), ultimately determine the long-term outcome and the degree of disability after rehabilitation (Cramer, 2008; Murphy and Corbett, 2009; Cramer et al., 2011; Di Pino et al., 2014, 2016; Di Lazzaro et al., 2015, 2016a; Bernhardt et al., 2017).

Direct contribution of lesion properties (e.g., side, location, etiology) to clinical outcome is limited and a significant role is played by alterations of brain areas beyond the lesion site (Dromerick and Reding, 1995; Pantano et al., 1996; Löuvbld et al., 1997; Miyai et al., 1997; Barber et al., 1998; Beaulieu et al., 1999; Chen et al., 2000; Vogt et al., 2012; Munsch et al., 2016; Dodd et al., 2017; Ernst et al., 2018; Pellegrino et al., 2019a). The latter mechanism is a solid concept in clinical and experimental neurology, introduced more than a century ago, and termed diaschisis (Carrera and Tononi, 2014).

The introduction of neuroimaging techniques allowing for whole brain functional mapping *in vivo* has demonstrated that behavioral impairments and potential recovery are linked to complex and distributed changes of brain functional activity and connectivity (Pellegrino et al., 2012, 2021; Silasi and Murphy, 2014; Burke Quinlan et al., 2015; Adhikari et al., 2017; Siegel et al., 2018). An overall rearrangement of brain function seems to occur in all stroke cases and is more pronounced in brain regions interconnected with the lesion site (Pellegrino et al., 2012; Di Lazzaro et al., 2014; Di Pino et al., 2014).

However, while a remarkable amount of research effort has been devoted to understanding changes of brain function, the effects of stroke on brain morphology, cortical thickness (CT) and cortical volume have not been fully characterized. It might be expected that regions beyond the lesion site may undergo cortical atrophy due to neuronal loss caused by disconnection (Carrera and Tononi, 2014; Di Pino et al., 2014). Conversely, brain regions may be expected to show cortical thickening of the areas participating during recovery via compensatory mechanisms, increased activity and consequently cortical plasticity (Di Pino et al., 2014). Alike functional changes, which show a dynamic evolution over time, potential changes of CT are expected to occur in a time period ranging from a few weeks to years after stroke lesion (Streitbürger et al., 2012).

The aim of this study is to systematically review the literature on the effects of stroke on CT beyond the lesion site and their potential relationship with clinical outcome in terms of sensory-motor function.

## Methods

The systematic review was conducted and reported according to the PRISMA guidelines (Moher et al., 2009), the protocol was registered on PROSPERO (https://www.crd.york.ac.uk/prospero/), with registration number: CRD42020200539.

We searched PubMed, Scopus, Web of Science, Cochrane, Embase databases using the following keywords: “stroke,” “cortical thickness,” “cortical atrophy,” “recovery,” and “brain lesion,” from inception until April the 16^th^ 2021. A detailed description of the search strategy can be found in Supplementary Materials (Appendix 1).

Articles were considered for inclusion only if:

- Enrolled subjects were stroke survivors, regardless of the nature and origin of the stroke (e.g., acute or chronic, ischaemic or haemorrhagic, cortical, subcortical, cortico-subcortical).
- Human adults were enrolled (>18 years of age).
- The relationship between CT and stroke functional recovery was explored, regardless of the experimental design.

Articles were excluded, if they enrolled animals or subjects affected from diseases other than stroke.

The literature search yielded a total of 821 results. After removing all duplicates, 662 articles were screened for inclusion by 2 independent review authors (AMC and LC), based on title and abstract, using the free online tool Rayyan (Ouzzani et al., 2016), for double blind selection. A third independent review author (AT) solved any disagreements. The full texts of the articles selected were independently reviewed by AMC and LC and the inclusion criteria were re-examined. Any disagreements were solved after discussion with a third reviewer (AT). Additionally, reference lists of the included articles were manually reviewed to increase the likelihood of identifying all relevant studies.

### Assessment of Risk of Bias in the Included Studies

Methodological quality of original articles was assessed with the Newcastle-Ottawa Scale (NOS) for non-randomized studies (Stang, 2010) and the Cochrane risk of bias assessment tool for Randomized Controlled Trials (RCTs) (Cumpston et al., 2019). The NOS includes three domains: the selection item refers to the methods for participants' enrolment, the comparability domain indicates how well the analysis of confounding factors was managed and, finally, the exposure domain refers to the ascertainment of the exposure. For RCTs, assessment was conducted following the guidelines stated by the Cochrane Collaboration in their Cochrane Handbook for Systematic Reviews of Interventions (Cumpston et al., 2019). We evaluated the following bias domains: (1) Random sequence generation, (2) allocation concealment (3) blinding of participants and personnel (4) blinding of outcome assessment, (5) incomplete outcome data and (5) selective reporting.

## Results

Search strategy identified 821 records from five electronic databases, and 3 more studies were included from manual search of the reference lists of the previously retrieved articles. We furthermore added 2 papers that were found while conducting an initial search on PubMed. After removing 164 duplicates and 646 studies with unrelated target topics, 16 studies remained for full-text review.

After the screening of full texts, 4 papers were excluded from the qualitative analysis since they did not fully meet the inclusion criteria.

**Figure 1:**
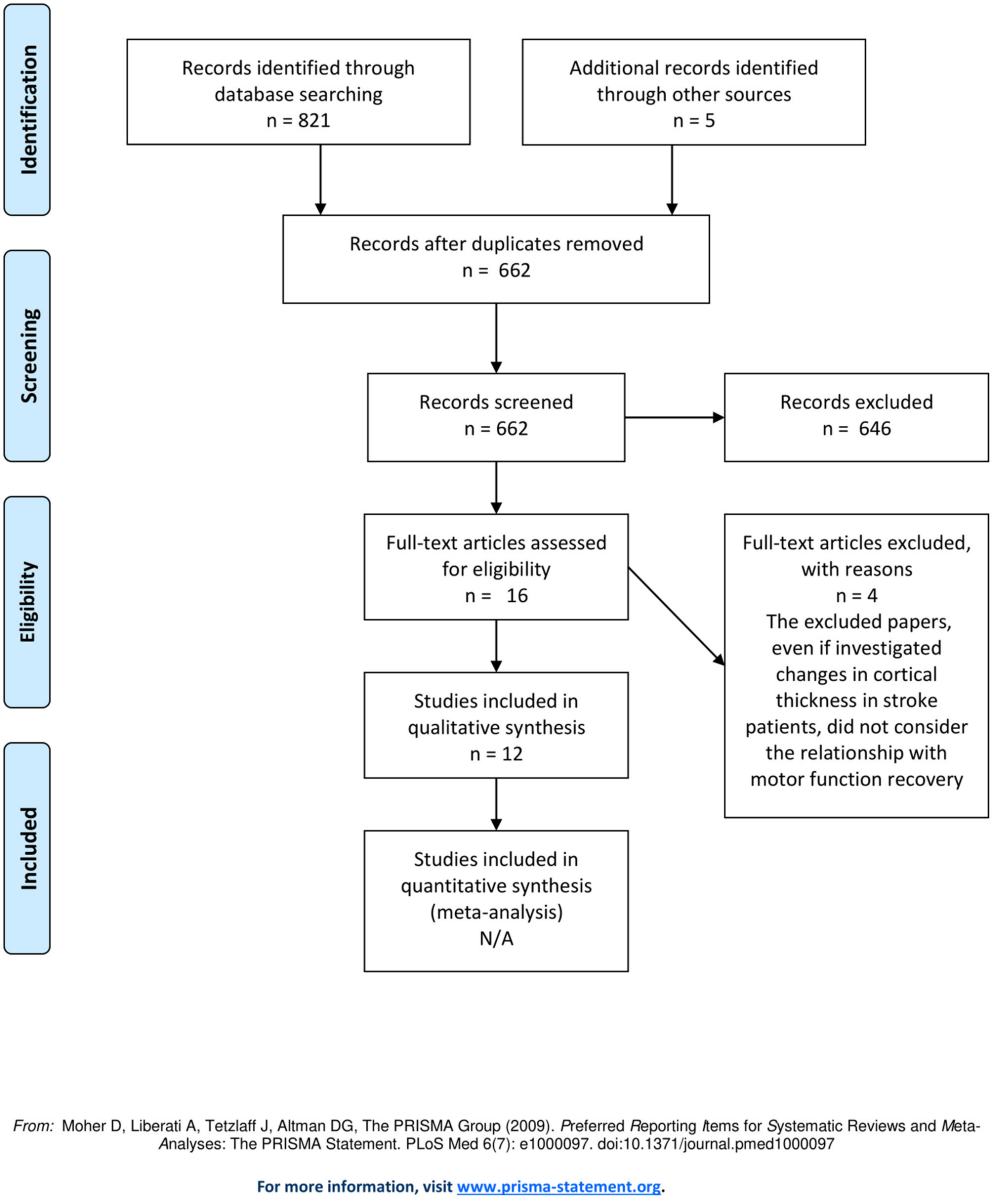
Flow diagram of the studies.

Finally, 12 studies were included for the qualitative analysis. Among them, 8 papers enrolled patients during the acute phase (<5 days post-stroke), whereas the remaining studies enrolled patients in the chronic phase (more than 6 months post stroke). The PRISMA flowchart of the review process is displayed in Figure 1. All relevant clinical, methodological and neuroimaging details are summarized in Table 1. We evaluated, if the retrieved data would be eligible for quantitative analysis (meta-analysis), which did not apply. The evaluation can be found in Appendix 2.

**Table 1:** Characteristics of included studies.

	**Aim**	**Type of study**	**Methods**	**Participant characteristics**	**Clinical measures**	**Imaging measures**	**Findings**
Buetefisch et al. (2018) Journal of Neurophisiology	To evaluate, if the affected hand function in chronic stroke is related to structural and functional reorganization of M1 and CST of the lesioned hemisphere.	Case-control	CT and FA served as measures of M1 and CST structure. Images were obtained on a Siemens 3T Trio scanner using a 12-channel head coil.	18 patients with chronic stroke involving either M1 and/or the CST. Cortical and subcortical stroke. Stroke data were compared with data from 18 age-matched healthy subjects.	MRC; modified Ashworth scale; JTT; peak acceleration of wrist extension movements for affected hand function; WMFT; MAL.	CT and FA as a measure of M1 and CST, respectively. CT measured using the Freesurfer software.	In chronic stroke patients with injury to M1 and/or CST an abnormally reduced M1 output is not related to impaired hand function.
Cai et al. (2016) Frontiers in Human Neuroscience	To investigate the potential structural cortical reorganization after subcortical stroke comparing findings on the acute phase (within 5 days from the stroke) and at 1 year.	Longitudinal study	1.5T MR scanner, T1W images.	11 right-handed patients with acute subcortical ischemic infarctions involving the basal ganglia regions.	NIHSS; MI	Gray matter volume obtained with VBM analyzed using a VBM8 toolbox implemented in the SPM software.	Structural reorganization of the contralesional cognitive-related cortices might contribute to motor recovery after subcortical stroke.
Chen et al. (2019) European radiology	To identify regions causally influenced by thalamic stroke and to determine the association between structural/functional alteration and somatosensory dysfunction.	Case-control study	3T MRI scanner. T1W; DTI; rsfMRI.	31 participants with chronic thalamic infarct and somatosensory dysfunction vs. 32 healthy controls.	NIHSS; FMA; BI; LMA	DTI; FA; rsFC. Cortical volume was calculated with the Freesurfer software.	Thalamic infarcts induce remote changes in the S1 and this network of abnormality underlies the cause of the sensory deficits.
Cheng et al. (2015) Journal of cerebral blood flow and metabolism.	To elucidate the impact of focal subcortical stroke lesions on CT.	Prospective MRI study with assessment at the acute phase and 3-month follow-up	3T MRI scanner. T1W and DTI images acquired.	12 patients with upper extremity paresis resulting from acute ischaemic subcortical stroke.	NIHSS; mRS; UEFM; ARAT; grip force	Combined white-matter tractography and semi-automatic measurement of CT using the Freesurfer software.	There is a specific impact of subcortical lesions on distant, yet connected cortical areas.
Cheng et al. (2020) Journal of cerebral blood flow and metabolism	To test if selective cortical atrophy of brain areas connected to subcortical stroke lesions is observable in the late chronic stage 1 year after stroke, specifically in contralesional, homologous brain areas.	Prospective MRI study with assessment at the acute phase and 1 year follow-up	3T MRI scanner. T1W and DTI images acquired.	18 patients with chronic subcortical stroke.	NIHSS; UEFM; mRS	Combined white-matter tractography and semi-automatic measurement of CT using the Freesurfer software.	Atrophy of remote cortical areas connected to single subcortical lesions remain prominent one year after ischemic stroke. Contralesional cortical atrophy is detectable in homologous cortical areas.
Gauthier et al. (2012) Stroke	1. to evaluate the relationship between chronic motor deficits in stroke and the degree of thinning in normal-appearing brain regions on MRI. 2. to see if regional gray matter thinning in chronic stroke patients before treatment is related to the magnitude of improvement in motor status after CIMT.	RCT	1.5T MRI or 3T MRI scanner. T1W MRI VBM to relate gray matter density (in brain areas without visible damage) to motor status of the paretic arm.	85 chronic stroke patients with mild-moderate motor deficit.	MAL; WMFT	Gray matter density with Voxel Based Morphometry (VBM) using SPM5 toolbox,	Pre-treatment: lower MAL and longer performances at WMFT correlated with reduced GM density in ipsi- and contralateral motor areas. Less improvement of WMFT and MAL following CIMT was predicted by reduced GM density in motor areas remote from the infarct.
Jones et al. (2016) Restor Neurol neurosc	To see how regional structural differences, including CT, may be associated with metabolic function after stroke.	Cross-sectional study	3T MRI scanner. Metabolic and structural (T1W MRI) assessment of the primary motor cortex using H1 magnetic resonance spectroscopy and CT measurement. Average CT in the precentral gyrus in both stroke (ipsilesional/contralesional) and control (non-dominant/dominant) groups was compared.	17 subcortical ischaemic stroke in the chronic phase (>6 months) and 11 neurologically healthy controls	WMFT	tNAA concenration Glx concentration precentral gyrus thickness CT measured with surface based cortical morphometry using Freesurfer.	Ipsilesional precentral gyrus thickness and tNAA concentration were associated with UE motor performance. Precentral gyrus thickness was significantly lower in the stroke group compared to the control group, and ipsilesional thickness in the stroke group was not significantly associated with UE motor performance.
Kraemer et al. (2004) The American society of neuroimaging	To assess post-ischemic brain.	Retrospective study	1.5T MRI. T1W MRI	10 patients suffering from a first acute cerebral ischemia in the territory of the middle cerebral artery.	ESS	T1W MRI, VGM	Delayed brain atrophy after acute ischemic stroke involved areas anatomically connected with the ischemic brain lesion, that was accompanied by a simultaneous improvement of the neurological deficit.
Liu et al. (2015) European journal of neurology	To assess the relationship between the spontaneous neuronal activity in the motor-related cortex and motor recovery.	Case-control study	3T MRI. T1W	22 patients with acute subcortical infarct and 22 healthy subjects	FMA; NIHSS	CT analysis combined with ALFF calculation; FMA.	Increased spontaneous neuronal activity of M1 area may contribute to early motor recovery in patients with subcortical infarction.
Liu et al. (2020) Neurology	Cortical thickness was measured over a 6 months period to investigate cortical reorganization after basal ganglia stroke.	Case-control study	3T MRI at 1–7, 14, 30, 90, 180 days post-stroke (T1-T2 and FLAIR)	33 patients with first episode basal ganglia stroke and 23 age-matched control participants	FMA	CT measured with the Freesurfer software.	Increased CT in the ipsilateral and contralateral hemisphere were seen in patient's group at six months post stroke. CT increase was uncorrelated with behavioral improvement or with the FMA at the baseline.
Sterr et al. (2013) Neuroimage: Clinical	To examine structural changes in the non-lesioned hemisphere of 31 patients with chronic stroke undergoing CIMT. It was assumed that CT would change with the intervention and that this change should be greater in CIMT.	RCT	3T MRI. T1-weighted; DWI	31 patients with moderate to severe chronic upper-limb hemiparesis of the left (*N* = 15) or the right (*N* = 16) arm following first ever stroke (14 CIMT, 17 NO CIMT)	MAL; WMFT	CT measured with the Freesurfer software.	Non-lesioned hemisphere analysis revealed an increase in CT after therapy with a cluster peak centered over the precentral gyrus, postcentral gyrus There was no correlation between treatment effect and FA in the non-lesioned hemisphere CT in the contralesional hemisphere is not altered by CIMT.
Yu et al. (2017) European Journal of Neuroscience	Gray matter atrophy co-existed with brain plasticity presenting with structural remolding and hyperperfusion in specific GM regions during stroke recovery.	Prospective	MRI scans on a 3T scanner in the acute phase and 6 month follow-up. MRI to detect the GM volume change and non-invasive ASL-MRI to quantify CBF change.	12 acute ischaemic stroke patients with pure subcortical lesions.	NIHSS; BI	GMV using SPM8, CBF	Decreased GMV: ipsilateral post-central gyrus, pre-central gyrus, precuneus, angular gyrus, insula, thalamus and cerebellum. Increased GMV: ipsilesional hippocampus, contralesional orbital gyrus and lingual gyrus. Decreased GMV in the anterior lobe of cerebellum was negatively associated with improvement of BI.

*M1, primary motor cortex; CST, cortico-spinal tract; CT, cortical thickness; FA, Fractional Anisotropy; MRC, Medical Research Council; JTT, Jebsen-Taylor Test; WMFT, Wolf Motor Function Test; MAL, Motor Activity Log; VBM, Voxel Based Morphometry; NIHSS, National Institutes of Health Stroke Scale; MI, Motricity Index; FMA, Fugl Meyer Assessment; BI, Barthel Index; LMA, Lindmark assessment; DTI, diffusion tensor imaging; rsFC, resting state functional connectivity; S1:somatosensory area; mRS, modified Rankin Scale; UEFM upper extremity Fugl-Mayer; ARAT, Action Research Arm Test; CIMT, Constraint induced movement therapy; SPM, Statistical Parametric Mapping; GM, gray matter; tNNA, total N-acetylaspartate; Glx, glutamate+glutamine; ESS, European Stroke Scale; ALFF, amplitude of low frequency fluctuation; VGM, voxel guided morphometry; ALS-MRI Arterial Spin Labeling MRI; CBF, cerebral blood flow*.

The cortical changes in regions different from the ischaemic lesioned area were the focus of the studies, together with investigating the related motor and sensory clinical outcomes. All the included studies found a variation of CT in brain areas beyond the stroke lesion both in the ipsilesional and contralesional hemisphere. Overall, the population included ischaemic stroke patients with lesions located prevalently in subcortical brain areas. As the included studies are rather heterogeneous, we report them below in two major groups: longitudinal studies and cross-sectional studies on patients with chronic stroke. The most relevant features and the methodology applied for each study are also reported in Table 1.

### Longitudinal Studies

#### Bilateral Distant Changes in Cortical Thickness

The study by Cai et al. (2016) longitudinally investigated the relationship between cortical volume extracted from MRI and clinical outcome measures, both administered in the acute phase (within 5 days) and at 1 year follow-up from stroke onset. The authors recruited 11 acute stroke patients with ischaemic subcortical stroke. Cortical volume was estimated from T1-weighted images acquired on a 1.5 MR scanner. Voxel Based Morphometry (VBM) was performed with the VBM8 toolbox for SPM (https://www.fil.ion.ucl.ac.uk/spm/) to measure the potential changes in gray matter volume (GMV) after stroke, knowing that GMV contains information about CT and cortical surface area. The National Institutes of Health Stroke Scale (NIHSS) and Motricity Index (MI) were used as clinical outcome measures. Authors found a correlation between changes in GMV (corrected for lesion volume) and the clinical variables, specifically demonstrating that a more pronounced cortical atrophy in the precentral gyrus ipsilateral to stroke lesion correlated with a worse clinical recovery. Conversely, higher GMV of the contralateral orbitofrontal cortex (OFC) predicted a better motor recovery.

A recent work longitudinally investigated bilateral CT changes in basal ganglia stroke patients and healthy controls over a time-span of 6 months with 3 Tesla MRI (Liu et al., 2020). CT changes were assessed at five time points (within 7 days post-stroke and again at 14, 30, 90, and 180 days after the event) in 33 patients. Patients were divided into 2 groups according to whether or not the lesions affected the functional motor regions of the striatum, defined using resting state f-MRI (the striatal motor group – SMD and the non-striatal motor group – N-SMD). Patients' motor function was assessed by the Fugl-Meyer scale performed before and after each MRI scan. Structural MRI data were processed using Freesurfer. Fourteen patients were classified into the SMD and 19 into the N-SMD. The cortical thickness changes were explored comparing the baseline and 180 days post stroke images across all stroke participants and a significant increase in cortical thickness was found both in the ipsilesional (frontal pole, superior frontal gyrus, medial prefrontal cortex) and contralesional hemisphere (frontal pole, precentral gyrus, ventrolateral prefrontal cortex, superior frontal gyrus, medial prefrontal cortex, superior and middle temporal gyri). Furthermore, the evolution of these changes throughout the five time points was explored and a significant increase in CT over time was seen in the ipsilateral and contralateral hemisphere in the patient group; in comparison, healthy control participants demonstrated no cortical thickness changes over time or at any point. Moreover, the SMD and N-SMD groups underwent different patterns of cortical reorganization after stroke. The CT differences between 7 and 180 days post-stroke did not correlate with improvement in Fugl-Meyer scores suggesting that CT changes may not have a linear relationship with motor improvement. Instead, the SMD group exhibited a larger motor impairment compared to the N-SMD group, which underlines the importance of considering the stroke location when assessing symptoms and recovery. These study findings showed that CT changes appear over time and in cortical areas beyond the lesion site and that these changes are due to post stroke reorganization, given the fact that healthy controls did not show increase in CT. This increase in CT might be a product of motor recovery and motor compensation even if no correlation with motor score was found.

Cheng et al. (2020) reported another longitudinal study with a follow up of 1 year, conducted on 18 patients with subcortical ischaemic stroke and upper limb paresis. In this study the authors' aim was to test, if selective cortical atrophy of brain areas connected to subcortical stroke lesions was observable in the late chronic stage after stroke specifically in the contralesional homologous brain areas. T1-weighted and DTI images were acquired on a 3T MRI scanner and only NIHSS, FMA and grip strength were considered as clinical outcome measures. Clinical and imaging data were collected at 3–5 days after stroke and after 1 year. CT was measured using the Freesurfer software from T1-weighted images. Results showed that cortical atrophy involved regions connected with the lesion on both the affected and unaffected hemispheres. At the 1 year follow up no relationship between CT and clinical outcome was found.

The hypothesis that after a subcortical stroke there is a secondary decay of CT in the motor areas related to the degree of motor function impairment was longitudinally investigated in another study by Liu et al. (2015). With their work, they aimed at confirming that, after an initial reduction of CT, there is a progressive increase in the neuronal activity in motor areas, which might be correlated with motor function recovery after stroke. To confirm this hypothesis CT analysis from structural T1 MRI and a functional parameter from resting state MRI were combined. 3T MRIs were performed in the acute phase (before day 7 post stroke), after 4 weeks and after 12 weeks. The population included 22 patients with acute ischaemic subcortical stroke and 22 healthy controls. The authors assessed patients' motor function with FMA and NIHSS before each MRI examination and the correlation between motor outcome and changes in the investigated imaging indices was additionally explored. Authors found that CT was reduced in the premotor cortex, supplementary motor cortex (SMC) and precuneus 12 weeks after stroke. Interestingly, an increase was found in mean CT in the supplementary motor cortex and the insula of the unaffected hemisphere. The thickening of the contralesional SMC was correlated with changes in FMA scores and patients with significantly increased CT in the contralesional SMC experienced a greater improvement in motor function.

Furthermore, Kraemer et al. (2004) aimed at assessing the relationships between cortical volume in the acute and chronic phases after ischemic brain lesion and clinical recovery. Their study retrospectively included 10 patients affected from ischaemic stroke in the territory of the middle cerebral artery assessed with the European Stroke Scale (ESS), which was administered in combination with their MRIs. T1-weighted images were acquired on a 1.5T MRI scanner at acute and chronic stage, but time of acquisitions were not standardized, thus it should be considered as limitation. To assess cortical brain volumes they used MRI voxel-guided morphometry. Results revealed a shrinkage in cortical volume in brain areas exceeding the ischaemic lesion. Remote changes were found in the ipsilateral hemisphere and, in several patients, in the contralateral one. Brain volume alterations were not related to age, recovery or time between scans. The authors hypothesized that paresis and reduced usage may lead to secondary changes in cortical brain volume.

The post-stroke recovery variability issue drove the group by Yu et al. (2017) to investigate the differences in the gray matter volume and cerebral blood flow in acute vs. chronic subcortical stroke in depth. The background hypothesis was that comparing brain reorganization at different times from onset could provide insights into the functional and anatomical bases of recovery after stroke. In the study design the time interval between scans was variable (ranging from 3 to 8 months) and imaging results were correlated with BI and NIHSS as outcome measures. Data from 12 acute stroke patients were analyzed, high resolution T1-weighted MRIs were acquired on a 3T scanner and data were processed with the voxel-based morphometry 8 (VBM 8) toolbox for Statistical Parametric Mapping 8 (SPM8). Cortical volume was significantly reduced from acute to chronic phase in several regions of the ipsilesional hemisphere such as the post central gyrus, the precentral gyrus, the insula, the precuneus, the angular gyrus, the thalamus, and the anterior cerebellar lobe. Also, cortical thinning was found in the anterior and posterior cerebellar lobes. Cortical thickening was seen in the ipsilesional hippocampus and the contralesional orbital and the lingual gyrus. Only the anterior cerebellar lobe atrophy in the contralesional hemisphere was negatively correlated with recovery. Interestingly, atrophy in the precentral gyrus, the main area of voluntary movements, had no effects on stroke recovery. Based on these findings, the authors hypothesized that the increased volume in specific brain regions may be a compensatory response in promoting recovery after stroke.

#### Ipsilateral Distant Changes in Cortical Thickness

Cheng et al. (2015) conducted a prospective study investigating the impact of subcortical stroke lesions on CT and on the recovery of upper limb function. Twelve acute ischaemic stroke patients with subcortical brain infarct were recruited and underwent a 3-month follow-up. The outcome of interest for functional recovery was the upper limb function, assessed by NIHSS, Action Research Arm Test (ARAT), Fugl-Mayer (FMA) and grip strength. Imaging data consisted of T1-weighted and DTI images acquired on a 3T MRI scanner combined with clinical examination (i.e., 3–5 days and 3 months after stroke). The study looked at CT changes between regions connected to the lesioned stroke area both in the affected and the unaffected hemisphere. Imaging data analysis was performed using the Freesurfer software package (Dale et al., 1999) (https://surfer.nmr.mgh.harvard.edu/). Clinical outcome measures of motor function improved in all patients. Results from cortical measurements showed significant cortical thinning involving the superior frontal gyrus and regions at the border of the supplementary motor area in the affected hemisphere, while non-significant changes were found in the unaffected hemisphere. The lesion size did not correlate with the CT changes. No direct effect of such thinning on upper limb motor performance was found and relative changes of clinical outcome measures were not significantly correlated with changes in CT. The authors speculated that the lack of correlation between CT changes and functional recovery could be due to the short follow-up period and the small sample size.

### Cross-Sectional and Chronic Stroke Studies

#### Bilateral or Contralesional Distant Changes in Cortical Thickness

Gauthier et al. (2012), aimed at evaluating the relationships between chronic motor deficit of the upper limb in stroke patients and the amount of thinning in brain regions not apparently affected. In addition, the study aimed at addressing the question of whether regional gray matter thinning can be related to the improvement of upper limb function after Constraint Induced Movement Therapy (CIMT). To answer the research question, they recruited 85 chronic stroke patients and assessed their upper limb function with the MAL and WMFT. Forty-four subjects underwent MRI scans and T1-weighted MR images were acquired on a 1.5T or a 3T scanner; cortical features were measured with voxel-based morphometry (VBM). The authors investigated the relationships between gray matter density and upper limb recovery to calculate a pre-treatment motor status and subsequently looked at the same measures in the CIMT group. The authors found that better clinical outcome, and in this case also clinical benefit after CIMT, was related to cortical thickness of the sensory-motor regions of the healthy hemisphere.

The relationship between CIMT and cortical morphology was also investigated by Sterr et al. (2013). The authors looked at CT variations in 31 patients with moderate to severe chronic stroke sequelae and compared CIMT vs. standard therapy. A 3T MRI scanner was used to acquire T1-weighted MR images that were processed for surface based analysis with Freesurfer. To assess motor function, the MAL and WMFT were administered before and after therapy. Results showed a significant improvement in all the clinical measures in the CIMT when compared with the control group. CT changed in the precentral and the post central gyrus of the non-lesioned hemisphere, no difference between groups was found. Furthermore, no significant cortical changes associated with modification in clinical outcomes were seen. The authors speculated that the variation of cortical properties reflected the use-dependent structural changes, such as the increase in synapses, dendrites, axonal spines and glial cells.

#### Ipsilateral Distant Changes in Cortical Thickness

Focusing on chronic patients, Buetefisch et al. (2018) aimed at evaluating, if impairment of hand function is related to structural and functional reorganization of the primary motor cortex (M1) and its corticospinal projections of the lesioned hemisphere. Eighteen patients with cortical and subcortical ischaemic infarction involving the primary motor area and the corticospinal tract (CST) were studied. Hand motor function was assessed with the Jebsen-Taylor test and a kinematic measure of hand function, i.e., the peak acceleration of wrist extension movements. Furthermore, the Wolf Motor Function Test (WMFT) and the Motor Activity Log (MAL) were used. Data from patients were compared with data from two groups of age-matched healthy subjects. It was found that the primary motor cortex of the affected hemisphere was thinner than the contralateral. A significant association between hand function and structural integrity of the primary motor system, as measured by the primary motor cortex thickness and cortical anisotropy of the cortico-spinal tract, was not found. A limitation of this study is that it was not possible to distinguish if the process leading to the changes in M1 CT is regenerative or degenerative since they didn't have a second set of measurements for comparison.

A definite focus on the neural substrate underpinning upper limb recovery after stroke was the aim of Jones et al. (2016) who used biochemical and MRI approaches within the framework of a case control study design. A total of 17 patients with chronic subcortical stroke and 11 healthy controls were recruited. The authors wanted to quantify anatomical and metabolic differences in the primary motor cortex and to look at their relationships with the hemiparetic arm function recovery in chronic stroke patients. Participants underwent 3T MRI scans and functional assessment. Cortical reconstruction and segmentation were performed with the Freesurfer software. Total N-acetylaspartate (t-NAA) and glutamate (Glx) concentrations were measured, as both are altered in chronic stroke and their level of change is related to arm impairment and CT (Cirstea et al., 2011, 2012). Upper limb motor function was assessed with the WMFT. A significant positive correlation was found between tNAA and M1 thickness for ipsilesional and contralesional hemispheres in the stroke group, and tNAA concentration explained a larger amount of variance in motor performance. The precentral gyrus thickness was significantly lower in the stroke group where ipsilesional thickness was not significantly associated with motor performance.

Finally, Chen et al. (2019) highlighted the somatosensory deficits in thalamic stroke with the aim to identify cortical regions causally influenced by the ischaemic damage and to determine the association between structural/functional alteration and somatosensory impairment. To fulfill this purpose, 31 patients with chronic sensory-motor impairments secondary to thalamic infarction and 32 age and sex-matched healthy controls were enrolled. Clinical examination included NIHSS, FMA, Barthel Index and Lindmark assessment. MRIs were acquired with a 3T scanner and cortical measures were extracted from the T1-weighted images employing Freesurfer. Results revealed decreased cortical volume in the ipsilesional primary somatosensory cortex demonstrating a link between alteration of the cortical volume and somatosensory impairment.

### Risk of Bias in Included Studies

**Figure 2:**
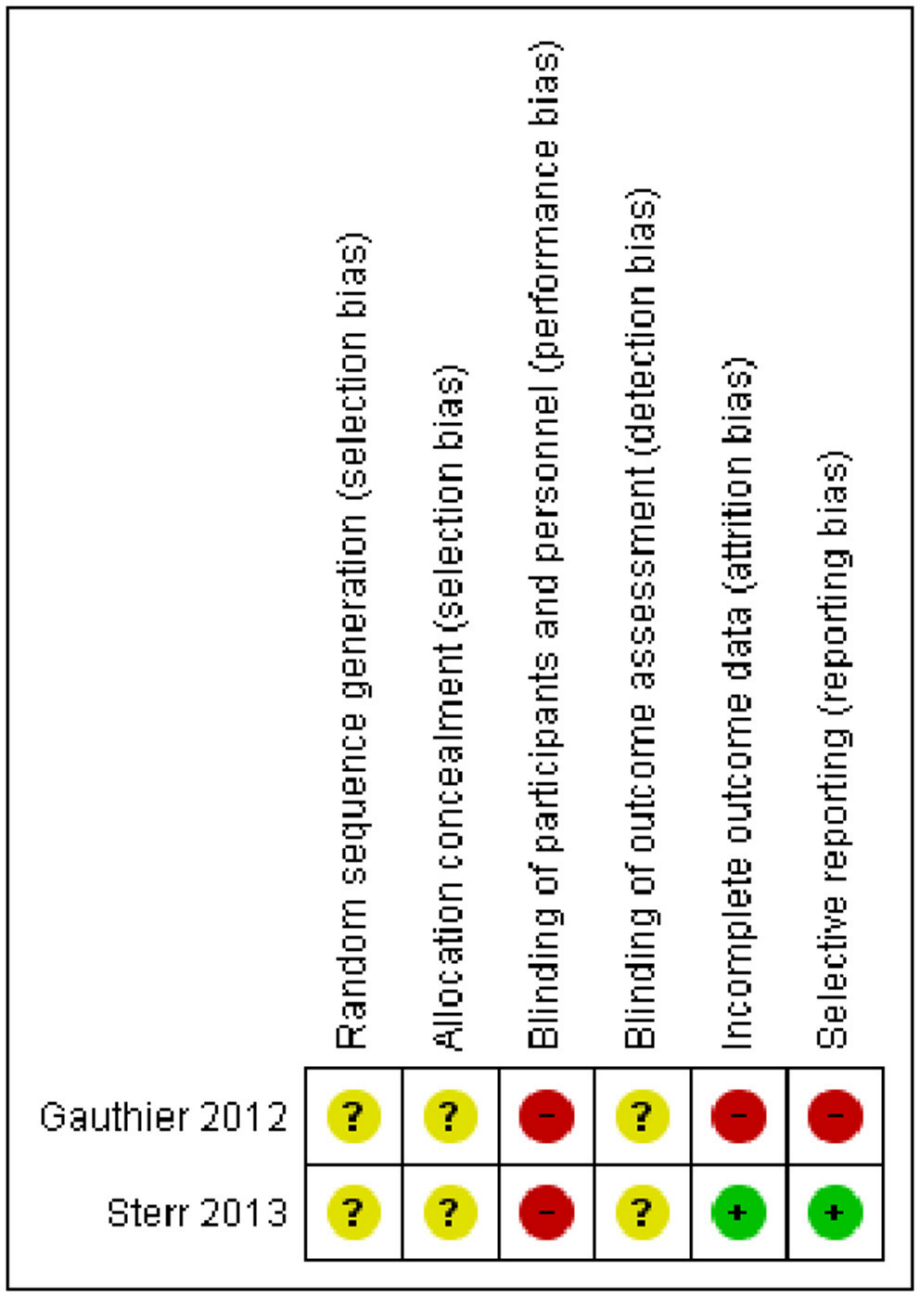
Risk of bias summary for Randomized Controlled Trials. Depicted are the review authors' judgements about each risk of bias item for the two included randomized controlled trials.

The methodological quality of the 10 non-randomized controlled studies was assessed by the Newcastle-Ottawa Scale and all studies received a NOS score <9, indicating a low methodological quality. The Risk of Bias Cochrane tool was used for methodological assessment of the 2 Randomized Controlled Trials and highlighted lack of information regarding randomization, allocation procedures and blinding of outcome assessment in all the studies. The detailed methodological assessment of non-RCTs is presented in Table 2, whereas the methodological assessment of RCTs is shown in Figure 2.

**Table 2:** Methodological quality of the included studies according to the Newcastle-Ottawa Scale (NOS) for case-control studies.

**References**	**Selection**	**Comparability**	**Exposure**	**NOS score**
Buetefisch et al. (2018)	***	**	***	8
Cai et al. (2016)	****	*	***	8
Chen et al. (2019)	***	**	***	8
Cheng et al. (2015)	****		***	7
Cheng et al. (2020)	****		***	7
Jones et al. (2016)	***		***	6
Kraemer et al. (2004)	**		***	5
Liu et al. (2015)	***	**	*	6
Liu et al. (2020)	***		**	5
Yu et al. (2017)	****		***	7

This table identifies “high” quality choices with a “star.” A study can be awarded a maximum of four stars (****) within the Selection category and a maximum of three stars (***) within the Exposure category. A maximum of two stars (**) can be given for Comparability. Studies can be divided into very high group quality (NOS score = 9) and lower quality group (score <9) for analysis.

### Excluded Studies

Four studies were excluded after reading the full text since they did not investigate the relation between sensory-motor recovery and CT (Brodtmann et al., 2012; Zhang et al., 2014; Duering et al., 2015; Werden et al., 2017). A detailed description of excluded studies is reported in the Appendix 3 of the Supplementary Materials.

## Discussion

In this review we aimed at identifying the effects of post-stroke changes in cortical thickness beyond the primary lesion location on sensory-motor outcomes. Twelve studies were included of which eight were focusing on subcortical lesions exclusively and four included patients with cortical and subcortical lesions. While seven studies could not show an association between CT and sensory-motor outcomes the remaining five found this correlation. Ten studies were case-control studies (Kraemer et al., 2004; Cheng et al., 2015, 2020; Liu et al., 2015, 2020; Cai et al., 2016; Jones et al., 2016; Yu et al., 2017; Buetefisch et al., 2018; Chen et al., 2019), while two were randomized controlled trials (Gauthier et al., 2012; Sterr et al., 2013).

Specifically, three studies show that a thinning of the ipsilesional pre- or postcentral gyrus was associated with worse motor and somatosensory recovery, respectively (Gauthier et al., 2012; Cai et al., 2016; Chen et al., 2019). This result was reproduced in a longitudinal design, a case control design and a cross-sectional design. This implies that (a) there might be a reduction in sensory-motor cortex volume in stroke secondary to primary lesion as a factor of time, (b) there might be a difference between cortical volume in stroke patients as compared to healthy controls in areas beyond the primary lesion, and (c) there might be differences in cortical reorganization after stroke beyond primary lesion, all contributing to the amount of recovery. Higher contralesional sensory-motor gray matter density was furthermore associated with improved motor ability (Gauthier et al., 2012). The study by Gauthier et al. enrolled subjects with up to 20 years chronic stroke phase. However, it is not clear, if patients with a higher contralateral gray matter density before the stroke have better motor outcomes after stroke or if this is a plastic effect related to stroke. Beyond the loss of gray matter as reflected in cortical thickness, stroke results in white matter loss that could influence remote functional networks via Wallerian degeneration (Wang et al., 2016). Loss of myelin in the precentral gyrus might play a crucial role in terms of functional recovery (Dubbioso et al., 2021).

Contralesional thickening in cortical areas beyond primary motor and sensory areas (i.e., OFC and SMC) was associated with improved motor recovery (Liu et al., 2015; Cai et al., 2016). Both studies were longitudinal and imply structural reorganization in contralesional areas beyond primary sensory-motor cortex. In one study decreased contralesional cerebellum thickness was correlated with worse motor recovery (Yu et al., 2017).

The remaining studies did not find an association between functional recovery and cortical thickness, although they have found changes in cortical thickness (Kraemer et al., 2004; Cheng et al., 2015, 2020; Jones et al., 2016; Buetefisch et al., 2018; Liu et al., 2020). Liu et al. (2020), while longitudinally evidenced CT increase ipsi- and contralesionally, did not find correlation with motor improvement. The authors hypothesize that this could possibly be due to the assessment scale they used. In fact, the FMA score represents a general assessment of limb function, and not accurately reflects changes in cognitive functions, which might be indirectly linked to stroke recovery. A relevant number of studies reviewed here, in fact, found a cortical thinning of the ipsilateral primary motor cortex (Gauthier et al., 2012; Liu et al., 2015; Cai et al., 2016; Jones et al., 2016; Yu et al., 2017; Buetefisch et al., 2018).

While the studies included here provide evidence of CT change due to stroke and a possible relationship with clinical outcome, the literature shows remarkable heterogeneities with respect to: patients' sample size (min/max = 10/85 patients, median = 18 patients), stroke location (purely subcortical vs. cortical & subcortical), study design (longitudinal, case control, randomized controlled trial), data analysis pipeline (voxel-based morphometry, surface-based analysis), and different magnetic field strength for MRI (2 1.5 Tesla, 9 3 Tesla, 1 mixed; please also check Table 1 for further details).

It is well-known that brain damage following a stroke is certainly the first cause of the complex sequelae occurring, but it is not the only factor defining the level of impairment so that long-term outcome depends upon lesion size and site, structural and functional reserve, and genetic pattern (Di Pino et al., 2014, 2016; Di Lazzaro et al., 2015, 2016b). Although studies in acute stroke report a correlation between lesion size and motor deficit, these deficits decrease in the chronic phase of stroke (Gauthier et al., 2012).

### Cortical Thinning and Its Possible Mechanisms

Some studies showed that the effect of the stroke lesion goes far beyond the motor system and the ipsilateral hemisphere, also involving the contralateral side (Cheng et al., 2015, 2020; Liu et al., 2015, 2020; Cai et al., 2016; Yu et al., 2017). In one of the studies examined (Cheng et al., 2020) the concept of “transcallosal diaschisis” was proposed to explain the cortical atrophy of contralateral homologous areas. It was suggested that isolated subcortical lesions could induce contralesional cortical degeneration through interneurons located at the ipsilateral hemisphere that induce apoptosis following loss of synaptic input (Carrera and Tononi, 2014). Post-stroke Wallerian or retrograde degeneration of fiber tracts originating from or projecting to the ischemic brain area has also been suggested as explanation of secondary atrophy (Kraemer et al., 2004; Cai et al., 2016; Cheng et al., 2020).

An alternative perspective on the mechanisms sustaining post-stroke changes in cortical thickness relates to the so-called “non-use cortical atrophy” corresponding to the effects of functional under-activation of primary and secondary motor areas due to motor impairment (Kraemer et al., 2004; Liu et al., 2015; Cheng et al., 2020).

### Cortical Thickening and Its Possible Mechanisms

A few studies have highlighted the presence of thickening of areas beyond the lesion site (Gauthier et al., 2012; Sterr et al., 2013; Liu et al., 2015, 2020; Cai et al., 2016; Yu et al., 2017), typically in the supplementary motor areas of the unaffected hemisphere. Interestingly, one of the studies included here found cortical thickening in the contralateral premotor cortex and supplementary motor area and a better motor performance for those having higher cortical thickness. Beyond their implication in fine motor planning and control, premotor and supplementary motor areas are remarkably involved in cognition at large (Tombini et al., 2009; Pellegrino et al., 2018c; Zangrandi et al., 2019), presumably highlighting how cognitive abilities are important for recovery of the motor function (Liu et al., 2015).

While this finding is not consistent over studies, it would be compatible with models predicting a variation of function (thereby structure) from regions that are spared from the stroke, and progressively work to compensate for the deficit. Animal studies support this hypothesis and have demonstrated that structural changes in neural cells such as increased neuronal sprouting, synaptogenesis as well as in non-neural elements (i.e., increase in glial cells and angiogenesis) occur over a period ranging from weeks to months after stroke (Liu et al., 2015). Moreover, a TMS experiment suggests that microglia play a crucial role in synaptic plasticity (Eichler et al., 2021). While remote neurones may degenerate secondarily to stroke due to a lack of structural connection and underuse, the targeted training of motor areas contralateral to the dominant hand has been shown to result in an increase in cortical thickness and cortical excitability (Sale et al., 2017).

TMS as stroke therapy has been applied to increase ipsilesional excitability or decreases contralesional excitability, addressing brain areas distant from the primary lesion. Processes of long-term potentiation or depression might be reflected in measures of brain structure and cortical thickness (Hebb, 2005; Dubbioso et al., 2015; Raffin et al., 2015; Smith and Stinear, 2016).

Correlations between CT changes and functional recovery were also seen in the study by Gauthier et al. (2012). Here gray matter density of areas beyond the infarct and involved in motor function, vision and cognition, positively correlated with clinical improvement suggesting an effect on more distributed neural networks, which together contributed to motor recovery in chronic stroke. When a correlation between structural changes and function was observed, cortical thinning, localized both ipsilaterally and contralaterally, was associated with worse recovery.

Additionally, motor function recovery was better for those having less cortical thinning in the ipsilateral motor area and cortical thickening in the contralateral areas (Liu et al., 2015). Not only cortical regions beyond the lesion change their structure and are relevant to recovery, indeed the degree of stroke recovery was negatively associated with contralesional cerebellar anterior lobule atrophy (Yu et al., 2017), which is associated to the ipsilesional motor cortex.

### Cortical Thickness as Clinical Marker

While this systematic review included all studies investigating the relationship between changes in structure and clinical sensory-motor outcome, the study situation remains inconclusive and is quite sparse. Stroke rehabilitation primarily aims at mitigating the clinical sequelae and restoring independence in activities of daily living (Langhorne et al., 2011). It focuses on techniques and strategies that can assess brain function and interfere with it (Cramer, 2004, 2008; Cramer et al., 2011; Pellegrino et al., 2012, 2018b). Furthermore, brain imaging and brain stimulation can be combined to achieve a temporally and spatially resolved investigation and interference with brain function (Giambattistelli et al., 2014; Assenza et al., 2015; Pellegrino et al., 2016a,b, 2018a,b, 2019b, 2021; Capone et al., 2017). Despite these advancements, there has been very little success in addressing and harnessing stroke recovery with neuroimaging techniques in clinical practice, most often because of their complexity and monetary costs in a real-life clinical setting beyond clinical research (Di Pino et al., 2014; Assenza et al., 2017; Gramigna et al., 2017; Machado et al., 2018; Cai et al., 2021). An exception is represented by structural MRI, which has entered clinical practice. Virtually all stroke patients without contraindications undergo an MRI with standard sequences, typically T1-weighted, T2-weighted, FLAIR and DWI (Masdeu et al., 2006; Jiang et al., 2010). Clinical data derived from structural MRI, if properly acquired and quantitatively analyzed, may contain precious information to better understand plasticity phenomena in stroke patients and provide hints about the clinical status and recovery from early stage after stroke as well as guide the rehabilitation strategy (Jiang et al., 2010).

This systematic review reveals that, as of now, only few studies addressed the relationship between CT beyond primary stroke lesion and sensory-motor outcome. While a huge corpus of studies exists investigating structure-function relationships e.g., in healthy aging subjects (Oschwald et al., 2019) or schizophrenic patients (Birur et al., 2017), the influence of CT secondary to stroke are poorly understood and require more attention, as well, as more systematic research in order to detect patterns of plasticity that might be indicative for recovery.

## Conclusions

The aim of this paper was to conduct a systematic review on the existing literature exploring changes in CT after stroke in regions beyond the main lesion and their potential relationships with functional recovery. We hypothesized that cortical changes beyond the lesion would occur, as predicted in animal models and human stroke models. Whether clinical outcome is associated with thinning of lesioned regions and connected areas or compensatory thickening of distant areas remains not properly investigated yet. There is evidence that, following a stroke, changes in CT occur both in regions directly connected to the main stroke lesion but also in the contralateral homologs and in the cerebellum. It has been hypothesized that anterograde and retrograde degeneration may explain these phenomena, but also metabolic changes may play a role. The studies performed so far are limited with regards to population type, sample size, procedure utilized to analyze data and to report results. Nonetheless, the importance of these results supporting the occurrence of structural plastic phenomena beyond the stroke lesion and their role in clinical outcome should not be underestimated. Further studies on larger patient samples need to be performed, taking into consideration a more comprehensive clinical assessment and addressing the positive/negative effects of different rehabilitation procedures. While such studies may appear demanding, their implementation could be easier, if accompanied by the improvement and standardization of clinical MRI acquisition procedures (volumetric acquisitions of T1-weighted images with good spatial resolution).

## Data Availability Statement

The original contributions presented in the study are included in the article/Supplementary Material, further inquiries can be directed to the corresponding author/s.

## Author Contributions

AC, LC, AT, and GP contributed to conception and design of the study. AC and LC performed data screening, extraction, and wrote the first draft of the manuscript. A-LS, AT, and GP wrote sections of the manuscript. AT performed data screening. All authors contributed to manuscript revision, read, and approved the submitted version.

## Funding

AC was supported by the Italian Ministry of Health Grant no. GR-2018-12367485. AT was supported by the Italian Ministry of Health Grant no. RF-2019-12371486. GP was supported by the Italian Ministry of Health Grant no. GR-2019-12368960.

## Conflict of Interest

The authors declare that the research was conducted in the absence of any commercial or financial relationships that could be construed as a potential conflict of interest.

## Publisher's Note

All claims expressed in this article are solely those of the authors and do not necessarily represent those of their affiliated organizations, or those of the publisher, the editors and the reviewers. Any product that may be evaluated in this article, or claim that may be made by its manufacturer, is not guaranteed or endorsed by the publisher.
