# Supplementary material for: Cortical Thickness of Brain Areas Beyond Stroke Lesions and Sensory-Motor Recovery: A Systematic Review
Source: Front Neurosci. 2021 Nov 3;15:764671. doi: 10.3389/fnins.2021.764671 (PMC8595399; doi:10.3389/fnins.2021.764671)
Supplement: Supplementary file 1 [file Data_Sheet_1.DOCX]

**APPENDIX 1. SEARCH STRATEGY**

**PUBMED**

**stroke:** ((((cerebrovascular disorders [MeSH] OR basal ganglia cerebrovascular disease [MeSH] OR brain ischemia [MeSH] OR carotid artery diseases [MeSH] OR intracranial arterial diseases [MeSH] OR intracranial arteriovenous malformations [MeSH] OR intracranial embolism and thrombosis [MeSH] OR intracranial hemorrhages [MeSH] OR stroke [MeSH] OR brain infarction [MeSH])) OR (brain injuries OR brain injury, chronic)) OR ((stroke* OR cva OR poststroke OR post-stroke OR cerebrovasc* or “cerebral vascular”))) OR (((cerebral OR cerebellar OR brain* OR vertebrobasilar) adj5 (infarct* OR isch?emi* OR thrombo* OR emboli* OR apoplexy)))) OR (((cerebral OR brain OR subarachnoid) adj5 (haemorrhage OR hemorrhage OR haematoma OR hematoma OR bleed*))))

**cortical**: "corticalization"[All Fields] OR "corticalized"[All Fields] OR "cortically"[All Fields] OR "corticals"[All Fields] OR "cortices"[All Fields] OR "cerebral cortex"[MeSH Terms] OR ("cerebral"[All Fields] AND "cortex"[All Fields]) OR "cerebral cortex"[All Fields]

**cortical**: "cortic*” OR "cerebral cortex"[MeSH Terms] OR ("cerebral"[All Fields] AND "cortex"[All Fields]) OR "cerebral cortex"[All Fields]

**thickness**: "thick"[All Fields] OR "thickness"[All Fields] OR "thicknesses"[All Fields])

**atrophy**: "atrophie"[All Fields] OR "atrophy"[MeSH Terms] OR "atrophy"[All Fields] OR "atrophied"[All Fields] OR "atrophies"[All Fields] OR "atrophying"[All Fields]

**recovery**: "recoveries"[All Fields] OR "recovery"[All Fields] OR “motor function” OR “recovery of motor function”

**brain**: "brain"[MeSH Terms] OR "brain"[All Fields] OR "brains"[All Fields] OR "brain's"[All Fields] OR “total brain volume” OR “infarct volume” OR “brain structure”

**lesion**: "lesion"[All Fields] OR "lesion's"[All Fields] OR "lesional"[All Fields] OR "lesions"[All Fields]

1. ((((cerebrovascular disorders [MeSH] OR basal ganglia cerebrovascular disease [MeSH] OR brain ischemia [MeSH] OR carotid artery diseases [MeSH] OR intracranial arterial diseases [MeSH] OR intracranial arteriovenous malformations [MeSH] OR intracranial embolism and thrombosis [MeSH] OR intracranial hemorrhages [MeSH] OR stroke [MeSH] OR brain infarction [MeSH])) OR (brain injuries OR brain injury, chronic)) OR ((stroke* OR cva OR poststroke OR post-stroke OR cerebrovasc* or “cerebral vascular”))) OR (((cerebral OR cerebellar OR brain* OR vertebrobasilar) adj5 (infarct* OR isch?emi* OR thrombo* OR emboli* OR apoplexy))) OR (((cerebral OR brain OR subarachnoid) adj5 (haemorrhage OR hemorrhage OR haematoma OR hematoma OR bleed*)))

2. "corticalization"[All Fields] OR "corticalized"[All Fields] OR "cortically"[All Fields] OR "corticals"[All Fields] OR "cortices"[All Fields] OR "cerebral cortex"[MeSH Terms] OR ("cerebral"[All Fields] AND "cortex"[All Fields]) OR "cerebral cortex"[All Fields]

3. "thick"[All Fields] OR "thickness"[All Fields] OR "thicknesses"[All Fields]

4. "atrophie"[All Fields] OR "atrophy"[MeSH Terms] OR "atrophy"[All Fields] OR "atrophied"[All Fields] OR "atrophies"[All Fields] OR "atrophying"[All Fields]

5. "recoveries"[All Fields] OR "recovery"[All Fields]

6. "brain"[MeSH Terms] OR "brain"[All Fields] OR "brains"[All Fields] OR "brain's"[All Fields]

7. "lesion"[All Fields] OR "lesion's"[All Fields] OR "lesional"[All Fields] OR "lesions"[All Fields]

8. #2 AND #3

9.#2 AND #4

10. #6 AND #7

11. #1 AND (#8 OR #9) AND #5 AND #10

**COCHRANE**

#1 "cerebrovascular disorders"

#2 "basal ganglia cerebrovascular disease"

#3 "brain ischemia"

#4 "carotid artery diseases"

#5 "intracranial arterial diseases"

#6 "intracranial embolism and thrombosis"

#7 "intracranial hemorrhages"

#8 stroke

#9 "vasospasm intracranial"

#10 "vertebral artery dissection"

#11 ((stroke* or poststroke or apoplex* or cerebral vasc* or brain vasc* or cerebrovasc* or cva* or SAH)):ti,ab,kw (Word variations have been searched)

#12 (((brain* or cerebr* or cerebell* or vertebrobasil* or hemispher* or intracran* or intracerebral or infratentorial or supratentorial or middle cerebr* or mca* or anterior circulation or basilar artery or vertebral artery) near/5 (isch?emi* or infarct* or thrombo* or emboli* or occlus* or hypoxi*))):ti,ab,kw (Word variations have been searched)

#13 (((brain* or cerebr* or cerebell* or intracerebral or intracran* or parenchymal or intraparenchymal or intraventricular or infratentorial or supratentorial or basal gangli* or putaminal or putamen or posterior fossa or hemispher* or subarachnoid) near/5 (h?emorrhag* or h?ematoma* or bleed*))):ti,ab,kw (Word variations have been searched)

#14 "cerebral cortex"

#15 ("cortical*" OR "cortices" OR ("cerebral" AND "cortex"))

#16 thick*

#17 atrophy

#18 atroph*

#19 recover*

#20 brain

#21 brain*

#22 lesion*

#23 #1 OR #2 OR #3 OR #4 OR #5 OR #6 OR #7 OR #8 OR #9 OR #10 OR #11 OR #12 OR #13

#24 (#14 OR #15) AND #16

#25 (#14 OR #15) AND (#17 OR #18)

#26 (#20 OR #21) AND #22

#27 #23 AND (#24 OR #25) AND #19 AND #26

**EMBASE**

**stroke:**

1. ‘cerebrovascular disease’/de or ‘brain disease’/de or ‘basal ganglion hemorrhage’/de or ‘brain hemangioma’/de or ‘brain hematoma’/de or ‘brain hemorrhage’/de or ‘brain infarction’/de or ‘brain ischemia’/de or ‘carotid artery disease’/de or ‘cerebral artery disease’/de or ‘cerebrovascular accident’/de or ‘cerebrovascular malformation’/de or ‘intracranial aneurysm’/de or ‘occlusive cerebrovascular disease’/de or ‘vertebrobasilar insufficiency’/de

2. (stroke* or poststroke or apoplex* or cerebral vasc* or brain vasc* or cerebrovasc* or cva* or SAH)

3. ((‘brain*’ or ‘cerebr*’ or ‘cerebell*’ or ‘vertebrobasil*’ or ‘hemispher*’ or ‘intracran*’ or ‘intracerebral’ or ‘infratentorial’ or ‘supratentorial’ or ‘middle cerebral artery’ or ‘MCA*’ or ‘anterior circulation’ or ‘posterior circulation’ or ‘basilar artery’ or ‘vertebral artery’ or ‘space-occupying’) near/5 (‘isch?Emi*’ or ‘infarct*’ or ‘thrombo*’ or ‘emboli*’ or ‘occlus*’ or ‘hypoxi*’))

4. ((‘brain*’ or ‘cerebr*’ or ‘cerebell*’ or ‘intracerebral’ or ‘intracran*’ or ‘parenchymal’ or ‘intraparenchymal’ or ‘intraventricular’ or ‘infratentorial’ or ‘supratentorial’ or ‘basal gangli*’ or ‘putaminal’ or ‘putamen’ or ‘posterior fossa’ or ‘hemispher*’ or ‘subarachnoid’) near/5 (‘h?emorrhag*’ or ‘h?ematoma*’ or ‘bleed*’))

**cortical**:

5. ("cortical*" OR "cortices" OR ‘cerebral cortex’/de OR ("cerebral" AND "cortex"))

**thickness**:

6. “tick*”

**atrophy**:

7. “atroph*” OR ‘atrophy’/de

**recovery**:

8. “recover*”

**brain**:

9. **“**brain*” OR ‘brain’/de

**lesion**:

10. “lesion*”

11. #1 OR #2 OR #3 OR #4

12. #5 AND #6

13. #5 AND #7

14. #12 OR #13

15. #9 AND #10

16. #11 AND #14 AND #8 AND #15

**SCOPUS**

1. **stroke**: ( TITLE-ABS-KEY (“cerebrovascular disorders” OR “basal ganglia cerebrovascular disease” OR “brain ischemia” OR “carotid artery diseases” OR “intracranial arterial diseases” OR “intracranial arteriovenous malformations” OR “intracranial embolism and thrombosis” OR “intracranial hemorrhages” OR stroke OR “brain infarction” OR “brain injuries” OR “brain injury, chronic” OR stroke* OR cva OR poststroke OR post-stroke OR cerebrovasc* or “cerebral vascular” OR cerebral OR cerebellar OR brain* OR vertebrobasilar W/5 infarct* OR isch?emi* OR thrombo* OR emboli* OR apoplexy OR cerebral OR brain OR subarachnoid W/5 haemorrhage OR hemorrhage OR haematoma OR hematoma OR bleed* AND ( LIMIT-TO ( DOCTYPE , "ar" ) ) AND ( LIMIT-TO ( LANGUAGE , "English" ) )
2. **cortical**: (TITLE-ABS-KEY ("cortical*" OR "cortices" OR "cerebral cortex" OR ("cerebral" AND "cortex")))
3. **thickness**: "thick*"
4. **atrophy**: "atroph*”
5. **recovery**: "recover*”
6. **brain**: "brain*"
7. **lesion**: "lesion*"
8. #2 AND #3
9. #2 AND #4
10. #6 AND #7
11. #1 AND (#8 OR #9) AND #5 AND #10

**WEB OF SCIENCE**

**1.stroke**:

#1 TS=(“cerebrovascular disorders” OR “basal ganglia cerebrovascular disease” OR “brain ischemia” OR “intracranial arterial diseases” OR “intracranial arteriovenous malformations” OR “intracranial embolism and thrombosis” OR “intracranial hemorrhages” OR stroke OR “brain infarction” OR “brain injuries” OR “brain injury, chronic” OR stroke* OR cva OR poststroke OR post-stroke OR cerebrovasc* or “cerebral vascular” OR cerebral OR cerebellar OR brain* OR vertebrobasilar near/5 infarct* OR isch?emi* OR thrombo* OR emboli* OR apoplexy OR cerebral OR brain OR subarachnoid near/5 haemorrhage OR hemorrhage OR haematoma OR hematoma OR bleed*)

**2.cortical**:

#2 TS=(cortical OR cortical* OR "cerebral cortex" OR cerebral AND cortex)

**3.thickness**:

#3 TS=(thick OR thickness OR thicknes*)

**4.atrophy**:

#4 TS=(atrophy OR atroph* OR atrophied OR atrophies OR atrophying)

**5.recovery**:

#5 TS= recovery OR recover*

**6.brain**:

#6 TS= brain OR brain* OR brain’s

**7.lesion**:

#7 TS=lesion OR lesion's OR lesion*

**8.** #3 AND #2

**9.** #4 AND #2

**10.** #7 AND #6

**11.** #1 AND (#8 OR #9) AND #5 AND #10
