# Supplementary material for: Cortical Thickness of Brain Areas Beyond Stroke Lesions and Sensory-Motor Recovery: A Systematic Review
Source: Front Neurosci. 2021 Nov 3;15:764671. doi: 10.3389/fnins.2021.764671 (PMC8595399; doi:10.3389/fnins.2021.764671)
Supplement: Supplementary file 2 [file Data_Sheet_2.DOCX]

**APPENDIX 2: QUANTITATIVE ANALYSIS OF INCLUDED STUDIES**

After qualitative synthesis, we collected quantitative data to carry out meta-analysis. We performed activation likelihood estimation (ALE) meta-analysis using Ginger ALE 3.0.2 ( <https://www.brainmap.org/ale/>) ^1^ to assess CT changes after functional recovery. ALE method considers brain activation coordinates as centers of probability distribution ^2^, thus each coordinate indicates that the brain activation is most likely located in that area, but it can also be in closed areas. ALE maps are then obtained by computing the union of activation probabilities across experiments for each voxel ^3^. In order to achieve sufficient power to detect smaller effects and to make sure that results are not driven by single experiments ^2^ ALE meta-analysis is recommended to include 17 to 20 experiments at least. In case that coordinates are reported in the Talairach space transformation into MNI space was performed by the icbm2tal function ^4^. Then, the coordinates and the number of subjects included in each study were used as input.

A total of 5 studies were eligible for meta-analysis, reporting foci coordinates (*x, y, z*) (Yu 2017, Zhang 2014, Chen 2019, Cheng 2015, Sterr 2013). Thus, we were not able to extract enough data to reach sufficient power for significant results of meta-analysis. Furthermore, all studies included in the quantitative meta-analysis assessed different imaging outcomes, therefore we were not able to achieve the minimum number of experiments needed to detect significant effects.

1. Eickhoff SB, Laird AR, Grefkes C, Wang LE, Zilles K, Fox PT. Coordinate-based activation likelihood estimation meta-analysis of neuroimaging data: A random-effects approach based on empirical estimates of spatial uncertainty. *Hum. Brain Mapp.* 2009;30:2907–2926.

2. Eickhoff SB, Nichols TE, Laird AR, et al. Behavior, sensitivity, and power of activation likelihood estimation characterized by massive empirical simulation. *NeuroImage*. 2016;137:70–85.

3. Turkeltaub PE, Eickhoff SB, Laird AR, Fox M, Wiener M, Fox P. Minimizing within-experiment and within-group effects in activation likelihood estimation meta-analyses. *Hum. Brain Mapp.* 2012;33:1–13.

4. Lancaster JL, Tordesillas-Gutiérrez D, Martinez M, et al. Bias between MNI and Talairach coordinates analyzed using the ICBM-152 brain template. *Hum. Brain Mapp.* 2007;28:1194–1205.
