# Supplementary material for: Cortical Thickness of Brain Areas Beyond Stroke Lesions and Sensory-Motor Recovery: A Systematic Review
Source: Front Neurosci. 2021 Nov 3;15:764671. doi: 10.3389/fnins.2021.764671 (PMC8595399; doi:10.3389/fnins.2021.764671)
Supplement: Supplementary file 3 [file Data_Sheet_3.docx]

**APPENDIX 3. DESCRIPTION OF EXCLUDED STUDIES**

The following papers were excluded after reading the full text articles since the association with functional recovery and CT changes was not investigated.

Broadtman et al., ^1^ conducted a longitudinal case-control study looking at the cortical thickness changes differences between an early stroke stage and 3 months later finding a decline of ipsilesional hippocampal and thalamic volume in the 3 months after stroke, but also an increase in contralesional areas known to be involved in motor recovery (paracentral, superior frontal and insular regions). Interestingly they found that the volumetric changes were more relevant in some subjects raising the possibility of using these volume changes to track individual decline. Furthermore they measured an increase in ipsilesional anterior cingulate thickness that is also of note knowing that cingulate regions are involved in motor learning and recruited with tasks of increasing complexity and that they are important in recovery after stroke affecting the motor regions.

Another longitudinal study by Duering et al., ^2^ investigated remote secondary effects on cortical areas connected to the infarct following up acute stroke patients at 6 months after stroke with structural MRI. They found that there was a significant degree of cortical thickness change between baseline and follow up in cortical areas connected to the stroke, but remote from it.

Werden et al., ^3^ examined structural MRI markers of brain aging including hippocampal volume, total brain volume and cortical thickness in ischaemic stroke patients with a first ever stroke or recurrent stroke and healthy controls. They found smaller brain volume in first ever stroke patients and recurrent stroke were associated with greater hippocampal volume loss.

In their paper Zhang et al., ^4^ explored the relationship between structural damage and functional reorganisation of M1 in patients with chronic subcortical stroke involving the motor pathway. Stroke patients - divided in two groups: partial recovery patients (PR) and complete recovery (CR) - when compared with healthy controls, showed reduced cortical thickness in the ipsilesional M1. The functional reorganisation was investigated through task fMRI and rsMRI and they compared functional activation of regions that showed significant decrease of CT in the stroke patients and controls. They found that PR stroke patients had an increased activation compared with healthy controls. The CR and PR groups exhibited stronger resting state functional connectivity (rsFC) compared with the control group. Interestingly CT analysis confirmed that M cortical thinning is observed also in stroke patients with CR of global motor function suggesting that motor recovery may be associated mainly with functional reorganization. No correlations between structural and functional changes were found. rsFC of the ipsilesional M1 could be used as a marker to predict post stroke recovery. The conclusion of this paper are very interesting, since they underline the fact that even if in those patients with subcortical stroke an alteration of the ipsilesional M1 structure has been seen, the M1 retain the potential of functional reorganisation and this is an important input to both increase the understanding of the role of ipsilateral M1 in poststroke recovery and to design rehabilitation strategies to restore ipsilateral M1 function after stroke such as transcranial stimulation of the M1 region.

**References**

1. Brodtmann A, Pardoe H, Li Q, Lichter R, Ostergaard L, Cumming T. Changes in regional brain volume three months after stroke. *J. Neurol. Sci.* 2012;322:122–128.

2. Duering M, Righart R, Wollenweber FA, Zietemann V, Gesierich B, Dichgans M. Acute infarcts cause focal thinning in remote cortex via degeneration of connecting fiber tracts. *Neurology*. 2015;84:1685–1692.

3. Werden E, Cumming T, Li Q, et al. Structural MRI markers of brain aging early after ischemic stroke. *Neurology*. 2017;89:116–124.

4. Zhang J, Meng L, Qin W, Liu N, Shi F-D, Yu C. Structural Damage and Functional Reorganization in Ipsilesional M1 in Well-Recovered Patients With Subcortical Stroke. *Stroke*. 2014;45:788–793.
